# Green Trust as a Mediator in the Relationship between Green Advertising Skepticism, Environmental Knowledge, and Intention to Buy Green Food

**DOI:** 10.3390/ijerph192416757

**Published:** 2022-12-14

**Authors:** Sara de Sio, Alessandra Zamagni, Giulia Casu, Paola Gremigni

**Affiliations:** 1 Department of Psychology, University of Bologna, Viale Berti Pichat 5, 40127 Bologna, Italy; 2 Ecoinnovazione Srl, Via della Liberazione, 6/c, 40128 Bologna, Italy

**Keywords:** green advertising skepticism, environmental knowledge, green claim trust, green purchase intention, green food

## Abstract

Changing individual habits towards greener choices is an essential ingredient in tackling the environmental crisis. Engaging in green behavior may improve psychological wellbeing. Although the intention to buy green products is widespread, the eco-friendly market is struggling to leave the ground. Greenwashing can increase skepticism towards green advertising, which in turn can hinder the intention to buy green products. Conversely, a better knowledge of environmental issues can promote a positive attitude towards environment and thus the intention to purchase green products. This study aimed to investigate if trust in green claims can mediate the relationships of green advertising skepticism and environmental knowledge with the intention to buy green food. An online survey was administered to 410 Italian consumers (63% female; 18–78 years). Our mediation model explained 23% of the variability in intention. Trust fully mediated the relationship between green advertising skepticism and intention to buy green food, while it partially mediated the relationship between perceived environmental knowledge and intention. Specifically, GAS was associated with lower INT through lower TR, whereas PEK was linked to higher INT though higher TR. The findings of this study can provide green market operators and policy makers with valuable information to encourage green food purchases.

## 1. Introduction

Decades of industrial human activity, characterized by an unsustainable consumption pattern, the use of non-renewable energy, and short-sighted capitalism, has led to an unprecedented situation of environmental degradation. Climate change, air pollution, mass migrations, deforestation, and biodiversity loss are only a few of the problems that this process caused. The ecologic transition has recently become a priority worldwide, although occidental countries have been well aware of the problem since the 1970s [1]. This situation poses a serious threat to the environment, human health, and society at large. Therefore, environmental interventions should involve every segment of the population, from government decisions to firms’ policies and individual consumer choices. Kates [2] pointed out that overpopulation and extreme consumerism are the two major drivers of the present environmental and ecological situation. A recent study reported that consumer household purchases were responsible alone for 40% of environmental damage [3]. Thus, the unplanned purchasing of goods can cause serious damage to the environment. Increasing the use of eco-friendly products could then be a possible solution to hinder this impact and stimulate more sustainable economic growth. Moreover, at the individual level, engaging in green behavior and perceiving consumer effectiveness, as the belief in one’s contribution to the environment preservation, may improve psychological wellbeing [4]. In a recent study, respondents to a self-administered survey declared that the consumption of organic food positively affected their subjective wellbeing and led to a better physical state, more positive emotions, and better social life [5]. Hence, adopting behaviors that have a lower environmental impact could benefit individuals’ health as well as the planet’s.

Green utilization implies that customers consider the ecological impact of buying, utilizing, and discarding items or services [6]. Such attitudes are in line with the objectives of the Green Deal [7] which focus on developing an ecological awareness to guide consumers’ choices. The theoretical framework most largely used to investigate factors that may influence consumers’ intention and behavior is based on the theory of planned behavior and the theory of reasoned action [8]. Such theories consider individuals as rational subjects who make systematic use of the available information to decide whether to adopt a certain behavior. In these theoretical models, intentions, which are cognitive representations of behavior adoption, are critical to the thought-action relationship. Therefore, assessing the antecedents of the intention to buy green products can be crucial to spread specific eco-friendly behavior.

In the last decades, consumers have started to show more and more concern and responsibility for the environment [9,10], and there is an ever-growing market for green and environmentally friendly products, ranging from conserving household appliances and green electric vehicles to green cosmetics [11]. Nevertheless, despite customers declaring to have environmental concern and positive attitudes towards green products, the market share of these items remains limited to just 7–8% of global commerce [12]. This phenomenon, whereby a highly positive attitude towards green purchase behavior is not followed by actual actions, is called “the green attitude-behavior gap” [13,14]. Although there is a vast literature on the subject, many researchers argue that this gap is still unexplained due to inconclusive results and a lack of systematic research [15].

Furthermore, a recent review on green purchase behavior [16] underpinned that in existing literature the focus is pointed mainly on green products in general, and not on specific product categories. Yet, the factors influencing purchasing intentions differ by product category, which is why the authors suggested keeping this in mind for future research [16]. Focusing on specific product categories could shed light on the often-disagreeing results on the topic, for example Nguyen et al.’s [17] and Sharma and Foropon’s [18] discord on the effect of perceived behavioral control on green purchase intention. Hence, following the suggestion of the above-mentioned review by Luthra and Deshwal [16], we focused our study on a specific category of green products, green food. Green food is defined as food that is protective and respectful of biodiversity and ecosystems, culturally acceptable, accessible, economically and nutritionally valuable, and made by optimizing natural and human resources [19]. Green food is also defined as a product with characteristics or production methods that cause less damage to the environment throughout its entire life cycle (from production to discarding) compared to other products of the same category [20]. The majority of the existing studies on green attitude and behavior consider the fields of pro-environmental manner and organic food consumption, more than green food [14]. Organic food refers to food produced through organic farming, a production system that uses natural manure and avoids synthetic fertilizers, pesticides, and chemicals as much as possible [21]. Although it has been suggested that the results of organic food can be used for comparison and reference with regards to green food [22], the adjectives “organic” and “food” describe two different concepts and shall not be used as interchangeably. We acknowledge that a misuse of the terminology is another factor that might hamper the research progression, and for this reason we have provided clear definitions of “organic food” and “green food”. An obstacle to the research progression could be indeed the lack of a common terminology regarding sustainable food. The words “green” and “sustainable” as well are often used interchangeably [8] even though there is no agreement on that in the literature. In this paper we focused specifically on green food based on a definition that distinguishes it from organic food.

### 1.1. Green Advertising and Greenwashing

Environmental advertising or green advertising can be defined as a message that tries to influence consumers’ cognitions, attitudes, and behaviors by promoting green features in the product’s whole lifespan, from production to distribution and disposal or recycling [1]. Such features can include actual characteristics of the product or stages of the production process itself that may reduce the environmental damage. In recent years, the rise of environmental concerns around the world has pushed many companies to improve their engagement and present their environmental efforts to the public [23,24]. Unfortunately, in some cases, green marketing claims do not reflect companies’ actual environmental conduct, exaggerate their efforts, omit their real environmental footprint, or make vague or false statements [25,26]. This phenomenon is called greenwashing [27]. Estimates on the diffusion of the greenwashing phenomenon seem to be discordant and unstable over time. An analysis of magazines at the beginning of the 1990s found that 58% of all green advertisements contained at least one misleading claim [19]. Subsequent studies found, on the contrary, that most green claims in magazines were truthful [11] and that misleading forms of green advertising were declining over time [28]. However, a more recent study highlights that reports of greenwashing have increased worldwide since “Dieselgate” [29] in September 2015, when the Volkswagen group was revealed to have falsified the emissions data of its cars, suggesting that the greenwashing phenomenon is still very subtle and diffused [30,31].

### 1.2. Green Advertising Skepticism and Trust in Green Claims

Since advertising is the main source of information on product characteristics, skepticism towards it can hinder the frequency of buying green products even among people concerned about the environment [32]. Previous research confirmed that consumers’ skepticism about green adverts was negatively related to their attitude towards green products [32,33]. The theory of reasoned action considers attitudes (i.e., the set of beliefs about the consequences of an action), together with subjective norms and perceived behavioral control, as predictors of intention, which in turn predicts behavior [34]. Hence, if consumers become suspicious of green claims, their attitude towards green products will get worse, and subsequent purchasing intention and behavior will become more unlikely. Green advertising skepticism is theorized as the negative cognitive component of consumers’ attitude toward green products consisting of the tendency not to believe the environmental information given by advertising [35,36]. Previous studies found that skepticism negatively influences purchase intentions by questioning the reliability, functionality, and truthfulness of green product claims [37,38]. On the contrary, trust in green claims is defined as consumers’ willingness to rely on an object grounded on its credibility, benevolence, and environmental performance [39]. Trust is related to a more positive attitude toward green products [40], so if consumers trust environmental claims about products, this will positively influence their purchase intention towards them [41]. Green trust has been theorized as a mediator between other constructs within the study of customers’ green purchase intention and behavior (e.g., between green brand image and green brand equity) [42]. However, it has not been studied yet as a mediator between green advertising skepticism and intention to buy green food. Therefore, for the current study, the following hypothesis was proposed.

**Hypothesis 1 (H1).**  *Trust in green claims mediates the relationship between green advertising skepticism and intention to buy green food*.

### 1.3. Environmental Knowledge

When customers are conscious of the environmental impact of items and build up a more informed attitude toward ecological protection, their awareness will affect their buying choices and may direct them to purchase green items [43]. Perceived environmental knowledge refers to an individual’s perceived knowledge about definitions, causes, and effects of environmental problems and about actions that would be necessary to address them [44]. It involves collecting information about specific or general aspects of environmental phenomena [45] and being aware of the collective responsibilities necessary for green development [46]. It has been suggested that more knowledge about environmental issues may promote positive attitudes toward the topic [44,46,47] and consequently green consumption intention and behavior [48,49,50]. A recent study [51] reported a significant positive relationship between both perceived green knowledge and green trust and customers’ intention to visit green hotels. Furthermore, green trust mediated the relationship of customers’ green knowledge with green visit intentions. Identifying the mediating role of green trust on the influential factors of customers’ green hotel visit intention has the merit of assisting the hotel business administrators to identify some of the underlying factors for choosing green hotels and to adopt business operations accordingly. We expected to replicate this finding shifting the focus from the intention to visit green hotels to the intention to buy green food. Therefore, the following hypothesis was proposed.

**Hypothesis 2 (H2).**  *Trust in green claims mediates the relationship between perceived environmental knowledge and intention to buy green food*.

### 1.4. The Present Study

**Figure 1:**
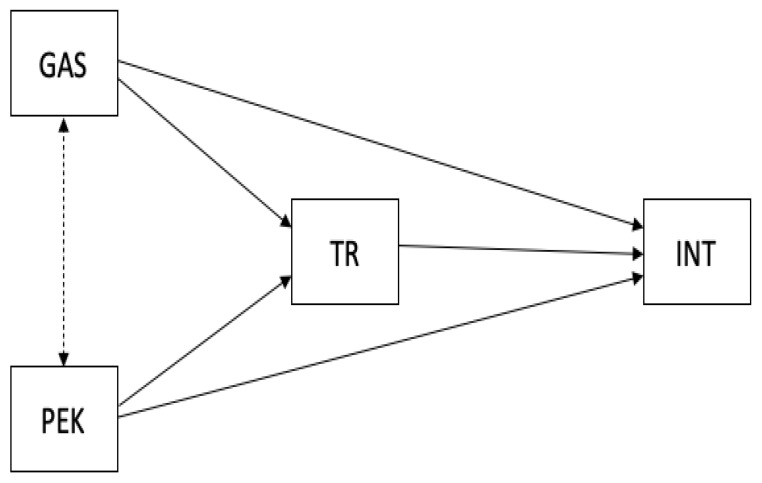
Conceptual framework of the study. GAS = green advertising skepticism; PEK = perceived environmental knowledge; TR = trust in green claims; INT = intention to by green products.

This study proposed a mediation model, as shown in Figure 1. To summarize, trust in green claims is argued to have mediating effects on the relationship between green advertising skepticism and intention to buy green food as well as in the relationship between perceived environmental knowledge and intention to buy green food. Furthermore, we assumed that gender could be a confounding variable related to two or more variables in the mediation model, partially explaining the relations between them, and thus it should be adjusted for in the mediation analysis. Previous studies have indeed found gender differences regarding environmental issues. For example, among fast food consumers, females were found to express a wish for green menus in terms of environmental impact more than males [52]. Among university students, women were found to be more concerned than men about green consumption [53].

## 2. Materials and Methods

### 2.1. Participants and Procedure

This study adopted a cross-sectional design. We recruited participants using an exponential, non-discriminative snowball sampling strategy through the researchers’ personal contacts in order to reach as many people as possible in a cost-effective way, following other authors’ example and suggestions [54,55,56]. The persons contacted throughout emails and private messages were sent a link to an anonymous online survey, asked to fill in the questionnaire and to invite their friends or acquaintances to do the same. Inclusion criteria to participate in the study were being 18 years or older and having, at least occasionally, the opportunity to purchase food. The survey was conducted from April to July 2022. A minimum sample size of 400 participants was defined a priori to reach enough power (0.80) to detect a mediated effect assuming small-to-medium-sized paths [57].

### 2.2. Measures

The survey included a demographic section (i.e., gender, age, education level, occupation, and household monthly income), an explanation of what “green food” stands for (Appendix A) and measures of the study variables. Regarding the constructs of the variables chosen, green advertising skepticism (GAS) was defined as the consumers’ tendency not to believe the claims made in green advertising and package labels [36]. Perceived environmental knowledge (PEK) was intended as how well-informed people thought they were about environmental issues and related necessary actions (e.g., production, packaging, symbols, recycling) [45]. Trust in green claims (TR) was intended as belief in eco-friendly companies and sellers’ honesty about the declaration of eco-friendliness of their products [58]. Intention to buy green food (INT) was defined as a prior, conscious decision to perform a certain behavior, in this case, purchasing green food [59].

To measure the constructs of interest, scales were taken from previous studies and mostly used as originally created, following Haws and colleagues’ advice [60] for deployment of scales in consumer research. Criteria for selecting the scales included connection to focal constructs, psychometric soundness (e.g., acceptable/good reliability), and a short length, to produce higher response and completion rates [61]. We adopted the four-item scale (total score 4–20; α = 0.79) developed by Mohr, Eroglu, and Ellen [36] to measure GAS. The six-item perceived knowledge scale [36] (total score 5–25; α = 0.86) was used to measure PEK. The four-item scale (total score 4–20; α = 0.94) developed by Voon, Ngui, and Agrawak [62] was used to measure TR. Finally, the three-item intention scale (total score 5–15) developed by Soyez [63] was used to measure INT. Table 1 shows construct, reference, and items content for each scale.

In the scales to measure TR and INT items were slightly modified by replacing “organic food” with “green food”. All items were rated using a 5-point scale (from 1 = “I completely disagree” to 5 = “I completely agree”). Scales were independently translated from English into Italian and then back-translated by two academic bilingual speakers. Validity and reliability were assessed in this study sample as well as potential common method bias [64].

### 2.3. Ethical Considerations

This study conducted a survey that involves human beings with the approval of the Ethical Research Committee of the University of Bologna (protocol number 0090636, 29 April 2022) according to ethical standards. Consumers voluntarily participated in the study and provided their consent by clicking on a button placed at the beginning of the online survey, right after an informed consent statement that described the study objective. This study ensured the anonymity, privacy, and security of the respondents.

### 2.4. Data Analysis

We conducted preliminary analyses including descriptive statistics, bivariate correlations between the study variables, and validity and reliability of the measures. Validity was tested using confirmatory factor analysis (CFA), the average variance extracted (AVE), and comparison of square root of the AVE for each construct with the correlation involving the constructs. Thresholds for the CFA goodness of fit indices were *χ²*/df < 3; root mean square error of approximation (RMSEA) <0.06; standardized root mean square residuals (SRMR) < 0.08, and comparative fit index (CFI) ≥0.95 [44]. AVE values should be >0.50 and its square root should be greater than the correlation between constructs [45]. Reliability was tested using McDonald’s *ω* and the composite reliability (CR) with acceptable values ≥ 0.70. Harman’s single-factor test was conducted to detect the problem of common method bias, which is considered to be present if the total variance extracted by one factor is >50% [65]. In addition, we examined the goodness of fit of a one-factor model of CFA.

A mediation analysis with the maximum likelihood (ML) estimation method was conducted to test the two hypotheses in a single model (Figure 1). A bootstrap resampling procedure with bias-corrected percentile and 1000 replications was used to confirm the significance of the indirect effects [66]. The analysis estimated total, indirect, and direct effects of the independent variables (GAS and PEK) on the outcome variable (INT) through the proposed mediator (TR).

For the interpretation of the results, we used both statistical significance (*p* < 0.05) and measures of effect size, with Pearson’s r of 0.10 considered small, 0.30 medium, and 0.50 large [67]. In bootstrap analysis, the effect was considered significant when the 95% confidence interval (CI) did not include zero. All statistical analyses were performed with JASP 0.16.3 software [68].

## 3. Results

### 3.1. Sample Characteristics and Descriptive Statistics

A total of 410 Italian adult consumers meeting the inclusion criteria agreed to participate; thus, the sample size was considered adequate. All of them stated that they personally bought food at least occasionally. The mean age of participants was 34.96 years (sd = 15.17; range 18–78), among them, 63% were females. According to the Italian educational system, 6% of the participants had a low level of education (5–8 years), 33% had a high school degree (12–13 years) and 61% had a university degree or masters/Ph.D. More than half of the participants (60%) were active community workers, 24% were undergraduate university students, 11% were student workers, and the remaining 5% were housewives, unemployed, or retired. With regards to household monthly income, 8% declared earning less than EUR 1000, 58% between EUR 1000 and 3000, 24% between EUR 3000 and 5000, and 10% more than EUR 5000 per month. The characteristics of the respondents are depicted in Table 2.

Descriptive statistics and bivariate correlations between variables are shown in Table 3. The correlation between GAS and PEK was nonsignificant. GAS correlated negatively with TR and INT, with moderate and small effect sizes, respectively. PEK correlated positively and weakly with TR and positively and moderately with INT. TR was positively, weakly correlated with INT. Moreover, a small effect was found in the association of gender (female coded 1 and male coded 0) with GAS (*r* = −0.12, *p* = 0.01) and INT (*r* = 0.11, *p* = 0.03). In particular, males tended to be more skeptical than females about green adverts, while females reported greater intention than males to purchase green food. Thus, we used gender in the mediation model as a confounding variable related to GAS and INT.

### 3.2. Characteristics of Measures

The results of the measurement model based on the four scales used indicated a good model fit with all indices meeting the required thresholds: χ2/df = 2.31; RMSEA = 0.06; SRMR = 0.07; and CFI = 0.97. All factor loadings were statistically significant, and AVE values were greater than the threshold value, except for GAS (Table 4). Furthermore, the square roots of AVE were greater than all Pearson correlations, thereby providing evidence for discriminant validity of the measures. Reliability was acceptable to very good for all the variables according to CR and *ω* values (Table 4). Thus, the measures in this study had acceptable validity and reliability.

Regarding Harman’s single-factor test, results of the unrotated solution of principal component analysis showed that four factors emerged explaining 70% of the variance and the first factor accounted for 30% of it that is less than the 50% threshold. Furthermore, results of one-factor model of CFA indicated a poor model fit with *χ*²/df = 12.99, RMSEA = 0.17, SRMR = 0.18, and CFI = 0.66. Therefore, we concluded that common method bias did not seriously compromise this study results, since the variance that was attributable to the measured method rather than to the constructs measured seemed to be acceptably low.

### 3.3. The Mediation Model

**Figure 2:**
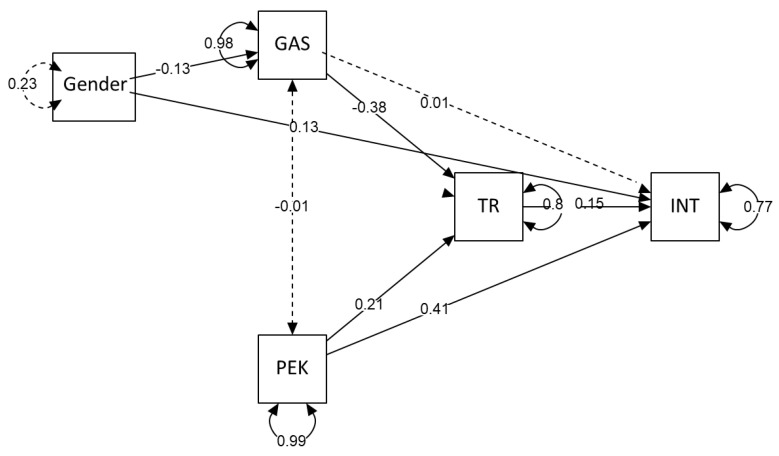
Results of hypothesis testing (N = 410). GAS = green advertising skepticism; PEK = perceived environmental knowledge; TR = trust in green claims; INT = intention to buy green food. Standardized parameter estimates are reported. Dotted lines indicate nonsignificant parameters.

The hypothesized mediation model tested (Figure 2) yielded a good fit, with *χ²*/df = 2.5; RMSEA = 0.06, SRMR = 0.03, and CFI = 0.99. It explained 23% of the variance in the values of INT and 20% of the variance of TR.

As shown in Table 5, the indirect effect of GAS on INT through the mediator TR was statistically significant as confirmed by the bootstrap analysis. As represented in Figure 2, GAS was significantly and negatively associated with TR, which in turn was significantly and positively associated with INT. Therefore, higher GAS was indirectly related to lower INT via a lower TR. The direct effect of GAS on INT without the mediator TR was nonsignificant, indicating full mediation. Thus, Hypothesis 1 was supported.

The indirect effect of the second independent variable PEK on the dependent variable INT through the mediator TR was statistically significant, as confirmed by the bootstrap analysis. As shown in Figure 2, PEK was positively and significantly associated with TR, which in turn was positively and significantly associated with INT. This means that higher levels of PEK were indirectly related to higher levels of INT through a higher TR. However, the direct effect (Table 5) was also significant, indicating that TR partially mediated the relationship between PEK and INT. Thus, Hypothesis 2 was supported.

## 4. Discussion

Green customers play a critical role in maintaining environmentally sustainable development over time, which can help preserve the health of the planet and entire societies. The present study aimed to investigate whether green advertising skepticism and perceived environmental knowledge were associated with the intention to buy green food through the mediation of trust in green claims. Although green skepticism and environmental knowledge were previously associated with green purchase intention [69,70] and green trust was used as a mediator between other variables and consumers’ purchase intention and behavior [41], no study has simultaneously considered all four dimensions together and with a specific focus on green food.

The results of correlation analyses showed that higher levels of skepticism about green claims were associated with lower intentions to purchase green foods, consistent with previous findings [70,71]. However, the results of the mediation analysis showed that when trust in green claims was entered into the model as a mediator, green advertising skepticism was not directly related to the intention to buy green food. Rather, it indirectly affected consumer green food purchase intention via the trust in green claims variable, which fully mediated this association, showing that consumers with high levels of green advertising skepticism tend to perceive green claims as more untrustworthy, and this in turn reduces their willingness to purchase green food. This result expands the body of knowledge about the underlying mechanisms of the negative association between green advert skepticism and green consumerism. According to previous literature [72], this can be the case in which advertising arguments are perceived as misleading and deceptive and, subsequently, people judge the information utility taken from them as low, developing advertising distrust. Information utility is how much information can help an individual make future decisions [73].

Results also showed that the positive association between perceived environmental knowledge and green purchase intention that was shown to be valid for green hotel visits [51] was confirmed for green food too. Thus, the more people know about environmental issues, their causes, and possible solutions, the more they will be willing to buy green food. The mediation analysis showed that trust in green claims partially mediates the relationship between perceived environmental knowledge and intention to buy green food. This means that environmental knowledge triggered trust in green food claims, positively affecting, as a result, green food-purchasing intentions. A possible explanation has been suggested in previous research, where green consumers (i.e., people who are aware of environmental problems and buy green products [74]) were found to trust green adverts more because they could distinguish between green and non-green foods or real green and greenwashed claims [75]. Despite the consistency of our finding with previous studies, we must also consider that they are discordant with other authors’ assumption that the more people are aware of green issues, the more skeptical they become towards green advertising because they are suspicious of greenwashing [76]. Noteworthy, GAS and PEK were unrelated in the present study, suggesting that skepticism in green advertising does not reflect or is not influenced by perceived knowledge of environmental issues [77]. On the other hand, a recent study [78] argued that even consumers with deep knowledge may not recognize vague greenwashing claims. Indeed, although they were more able than less informed individuals to detect false textual claims in a controlled experiment, this ability was inhibited when a nature-evoking image was added to a false claim. The authors concluded that environmental knowledge alone cannot be seen as a shelter from greenwashing, and future research must also consider other predictors and implicit mechanisms.

The overall findings of the present study were in line with the theory of reasoned action and the theory of planned behavior [8], which consider individuals as rational in deciding whether to adopt a certain behavior. Although the theory of planned behavior (TPB) [8] is still the most-used theoretical framework in the research field of green purchase intention and behavior [10], Danner and Thogersen [79] described two possible pathways in which green purchase decision-making occurs. In the first one, as posited by the TPB, the consumer chooses consciously what to buy when enough motivation, ability, and opportunity are present. In the second one, when these three factors are lacking, the purchase action will be guided by an automatically activated attitude, as theorized by Fazio [80]. Hence, it is reasonable to think that every consumer faces both the pathways theorized by Danner and Thogersen [79], when purchasing green food. This study focused on factors that can promote green purchase behavior in the first, more consciously-controlled pathway. However, future research should consider integrating the TPB with other models able to take into consideration behavioral automatisms.

The contribution of the present study to the theory, though, remains relevant as addressing factors that can promote green purchase intention in a consciously-controlled pathway could still help us understand more about the mechanisms that drive green purchase behavior. Moreover, the novel finding of the independence between knowledge and skepticism could help disprove the idea of the cultured skeptical consumer.

### Limitations

A number of possible sources of bias must be acknowledged in this study. First, the snowball sampling strategy used, although cost-efficient, may have introduced bias by missing out isolated members of the community or skewing towards subgroups of people who share the same characteristics or interests. Is it possible, in fact, that only people already interested in environmental issues were motivated to fill in the online survey, leading to a selection bias [81]. For this reason, caution should be used to generalize our findings and future research should consider using a random sample representative of the general population. Second, the exclusive use of self-reported instruments could have inflated the associations between variables because of the common method of assessment. Although data did not indicate evidence of severe common method bias, future studies should integrate information from multiple sources. Third, the scale used to measure green advertising skepticism had quite good psychometric characteristics, but its AVE value was under threshold, indicating a less than optimal convergent validity. Thus, a revision of this scale is needed. Fourth, in this study we used only a measure of self-perceived environmental knowledge, which refers to a different construct than factual knowledge. Although it has been suggested that measures of perceived knowledge may better assess an individual’s actual understanding of an issue [82], it would be relevant using both types of assessment in future research. Furthermore, it was suggested by an anonymous reviewer that we include aspects related to knowledge about green food eco-labeling in the PEK scale. Although it has been recognized that ecolabels can positively affect purchase intentions and selection of green products [80], this may represent a possible obstacle to our aim to assess this aspect regarding green food in particular, because self-declarations by producers are available, in many cases, covering only the carbon footprint of products, while current European ecolabels do not cover food products. However, it is worth mentioning that, as part of the Farm to Fork Strategy [83] the European Commission is working towards the setting-up of a sustainable food labelling framework, to assess and improve the eco-sustainability of food products and provide useful information to help consumers make informed choices. Future research on the topic should explore and integrate this aspect.

Fifth, the outcome of this study was the intention to purchase green food. Although theories of reasoned action and planned behavior consider intention a predictor of action, in the field of green purchase behavior a gap has been detected between intention and purchasing behavior [13]; therefore, actual green purchase behavior should be objectively measured in future research. Finally, although the proposed mediation model provided some information about the directionality of the relationships among the study variables, the cross-sectional design of the study did not allow to draw causal conclusions. Future studies should therefore consider the use of longitudinal data to more accurately examine the nature of the relationships among the variables.

## 5. Conclusions

To the best of the researchers’ knowledge, this study is the first attempt to examine a mediating role of trust in green claims in the relationship between skepticism towards green adverts or perceived ecological knowledge and intention to purchase green food. In spite of its limitations, this study identifies mechanisms that would benefit from further assessment. Based on previous research suggestions [16,73], we focused on intentions to buy a specific green product, green food. This helped to avoid possible confounding effects given by considering green products as indistinct elements of a unique category.

Within the theoretical framework adopted in this study, intentions are critical to the thought–action relationship; therefore, assessing the antecedents of the intention to buy green food can be crucial to spread specific behavior such as adopting a green diet. Industrial livestock production is responsible for a considerable amount of the ecological footprint of humans in terms of resource utilization and pollution [84]. Moreover, Western meat overconsumption is increasingly associated with food-related diseases like obesity, diabetes, or cardiovascular diseases [85], and it is proven that adopting an energy-balanced, low-meat diet can led to large reductions in premature mortality [86]. Hence, alternative food consumption patterns are needed, both from an environmental and a health perspective and would lead to profound improvements in our individual, national, and environmental health.

Based on empirical findings, this study provides suggestions for marketers and businesses that want to offer more efficient and targeted advertising on green foods. Marketers could consider green advertising skepticism and environmental knowledge as separate and unrelated but useful dimensions in designing more reliable and convincing adverts. They should consider as well the positive relationship observed between perceived environmental knowledge and positive intentions toward green purchase. It could be possible, for instance, to educate consumers about the convenience of buying ecologically safe products by placing reliable environmental facts and their related sources in advertisements and/or on the products packages. Moreover, government agencies and policy makers could consider these elements when developing interventions to raise public awareness of the individual and collective benefits of buying green foods and adopting a green diet. The findings of this study also provide implications for individual consumers. Individuals could improve their environmental awareness, their ability to distinguish between true and greenwashed adverts, and their belief that each consumer can help safeguard the environment. Engaging in green food consumption can indeed promote individual wellbeing and create value for the entire society.

## Figures and Tables

**Table 1:** Constructs, references, and items of measures.

Constructs and Reference	Items
Green advertising skepticism (GAS) [36]	Most environmental claims made on package labels or in advertising are true. (R)
	2. Because green claims are exaggerated, consumers would be better off if such claims in advertising were eliminated.
	3. Most environmental claims on package labels or in advertising are intended to mislead rather than to inform consumers.
	4. I do not believe most environmental claims are made on package labels or in advertising.
Perceived environmental knowledge (PEK) [36]	I know when I buy products and packages that are environmentally safe.
	2. I know more about recycling than the average person.
	3. I know how to select products and packages that reduce the amount of waste ending up in landfills.
	4. I understand the environmental phrases on product packages. 5. I am confident that I know how to sort my recyclables properly.
	6. I am very knowledgeable about environmental issues.
Trust in green claims (TR) [62]	I trust that those selling green food are honest about the ecological nature of their products.
	2. I trust that eco-friendly companies comply with environmental standards.
	3. I trust the green certification logo on green food labels.
	4. I trust the information on green food labels.
Intention to buy green food (INT) [63]	If I buy groceries next time, I will also buy green food.
	2. In the future I am going to buy green food.
	3. I intend to buy green food next time.

(R) = reverse item.

**Table 2:** Characteristics of participants.

Characteristic		Frequency	Percent
Education	Low level	24	5.85%
	High school degree	135	32.93%
	University degree	251	61.22%
Occupation	Student	98	23.90%
	Student worker	46	11.22%
	Active worker	247	60.24%
	Unemployed	19	4.63%
Income	<EUR 1000	31	7.56%
	EUR 1000–3000	239	58.29%
	EUR 3000–5000	97	23.66%
	>EUR 5000	43	10.49%

**Table 3:** Means, standard deviation, and Pearson’s correlations (N = 410).

	Mean (SD)	Range	1	2	3
1. GAS	10.48 (3.06)	4–19	-		
2. PEK	21.54 (5.04)	6–30	0.00	-	
3. TR	14.14 (3.28)	4–20	−0.40 **	0.21 **	-
4. INT	11.98 (2.95)	3–15	−0.09 *	0.44 **	0.26 **

Note. GAS = green advertising skepticism; PEK = perceived environmental knowledge; TR = trust in green claims; INT = intention to buy green food. * *p* < 0.05. ** *p* < 0.001.

**Table 4:** Psychometric characteristics of measures (N = 410).

Variable	Item	Loading	AVE	CR	McDonald’s *ω*	95% CI
GAS	G1	0.40 *	0.45 (0.67)	0.74	0.75	0.71–0.79
	G2	0.45 *				
	G3	0.77 *				
	G4	0.91 *				
PEK	P1	0.71 *	0.51 (0.71)	0.85	0.84	0.81–0.87
	P2	0.35 *				
	P3	0.78 *				
	P4	0.75 *				
	P5	0.76 *				
	P6	0.78 *				
TR	T1	0.72 *	0.70 (0.84)	0.90	0.90	0.89–0.92
	T2	0.82 *				
	T3	0.91 *				
	T4	0.88 *				
INT	I1	0.92 *	0.85 (0.92)	0.95	0.94	0.93–0.95
	I2	0.91 *				
	I3	0.94 *				

Note. GAS = green advertising skepticism; PEK = perceived environmental knowledge; TR = trust in green claims; INT = intention to buy green food. Square roots of AVE are reported in parentheses. * *p* < 0.001.

**Table 5:** Results of the mediation model.

Effects	β	SE	z	*p*	95% CI lb	95% CI ub
Total						
GAS→INT	−0.05	0.04	−1.15	0.25	−0.14	0.04
PEK→INT	0.44	0.04	9.97	<0.001	0.35	0.52
Indirect						
GAS→TR→INT	−0.06	0.02	−2.89	0.004	−0.10	−0.02
PEK→TR→INT	0.03	0.01	2.58	0.010	0.01	0.06
Direct						
GAS→INT	0.006	0.05	0.13	0.90	−0.08	0.10
PEK→INT	0.41	0.05	9.12	<0.001	0.32	0.49

Note. GAS = green advertising skepticism; INT = intention to buy green food; PEK = perceived environmental knowledge; SE = standard error; CI = confidence interval; lb = lower bound; ub = upper bound. Delta method standard errors, bias-corrected bootstrap confidence intervals.

## Data Availability

Although the data are not publicly available due to privacy and ethical restrictions, they are available from the corresponding author upon reasonable request.

## Appendix A

Definition of “Green food”: Since this research concerns green products, we will try to offer you a definition of this concept. A green product has characteristics or production methods that cause less damage to the environment throughout its life cycle (from production to end of life) compared to other products of the same category. For example, it uses renewable energy sources, non-toxic and/or biodegradable substances, is grown with organic methods (if it is a food product), produced locally, is packaged with recyclable materials, etc. In this research we will use the concepts of “green” and “eco-sustainable” as synonyms.
